# End-of-Life Recycling Options of (Nano)Enhanced CFRP Composite Prototypes Waste—A Life Cycle Perspective

**DOI:** 10.3390/polym12092129

**Published:** 2020-09-18

**Authors:** Fotini Petrakli, Anastasia Gkika, Alexandra Bonou, Panagiotis Karayannis, Elias P. Koumoulos, Dionisis Semitekolos, Aikaterini-Flora Trompeta, Nuno Rocha, Raquel M. Santos, Guy Simmonds, Glen Monaghan, Giorgio Valota, Guan Gong, Costas A. Charitidis

**Affiliations:** 1 IRES—Innovation in Research & Engineering Solutions, Rue Koningin Astritlaan 59B, 1780 Wemmel, Belgium; fpetrakli@innovation-res.eu (F.P.); agkika@innovation-res.eu (A.G.); abonou@innovation-res.eu (A.B.); karayannisp@innovation-res.eu (P.K.); 2 RNANO Lab.—Research Lab of Advanced, Composite, Nano-Materials & Nanotechnology, School of Chemical Engineering, National Technical University of Athens, GR-15773 Zographos Athens, Greece; diosemi@chemeng.ntua.gr (D.S.); ktrompeta@chemeng.ntua.gr (A.-F.T.); charitidis@chemeng.ntua.gr (C.A.C.); 3 INEGI—Institute of Mechanical Engineering and Industrial Management & LAETA—Associated Laboratory for Energy, Transports and Aeronautics, FEUP Campus, Rua Dr. Roberto Frias, 400, 4200-465 Porto, Portugal; nrocha@inegi.up.pt (N.R.); rmsantos@inegi.up.pt (R.M.S.); 4 AP&M—Anthony, Patrick and Murta Exportacao, Estrada Nacional 120-Falfeira—Lagos, 8600-308 Lagos, Portugal; simmonds.guy@gmail.com; 5 GSG—Global Safe Guard Ltd., 2 Longhorsley, Morpeth NE65 8RX, UK; info@globalsafeguard.com; 6 Brembo S.p.A, CURNO (Bergamo)—Via Brembo, 25, 24035 Curno, Italy; Giorgio_Valota@brembo.it; 7 RISE SICOMP AB, Fibervägen 2, 943 33 Öjebyn, Sweden; guan.gong@ri.se

**Keywords:** carbon nano tubes (CNTs), carbon fibre reinforced polymer composite (CFRP), recycling, sustainability, end-of-life (EoL), carbon fibres (CFs)

## Abstract

Life cycle assessment is a methodology to assess environmental impacts associated with a product or system/process by accounting resource requirements and emissions over its life cycle. The life cycle consists of four stages: material production, manufacturing, use, and end-of-life. This study highlights the need to conduct life cycle assessment (LCA) early in the new product development process, as a means to assess and evaluate the environmental impacts of (nano)enhanced carbon fibre-reinforced polymer (CFRP) prototypes over their entire life cycle. These prototypes, namely SleekFast sailing boat and handbrake lever, were manufactured by functionalized carbon fibre fabric and modified epoxy resin with multi-walled carbon nanotubes (MWCNTs). The environmental impacts of both have been assessed via LCA with a functional unit of ‘1 product piece’. Climate change has been selected as the key impact indicator for hotspot identification (kg CO_2_ eq). Significant focus has been given to the end-of-life phase by assessing different recycling scenarios. In addition, the respective life cycle inventories (LCIs) are provided, enabling the identification of resource hot spots and quantifying the environmental benefits of end-of-life options.

## 1. Introduction

**Figure 1:**
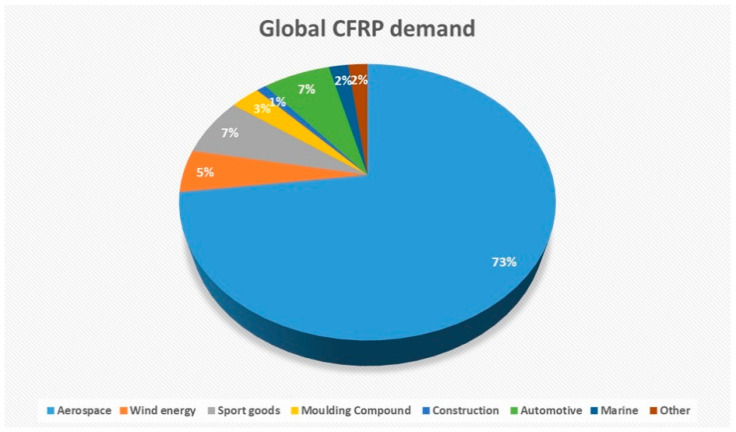
Global demand based on sales (2018) [20]. CFRP, carbon fibre reinforced polymer.

Composites science and technology are developing rapidly, providing new material solutions, addressing the requirement for high performance [1]. Composites have the ability to be tailored for specific end uses; they offer superior properties such as high strength to weight ratios, high flexural and impact strengths, corrosion and heat resistance, durability, dimensional stability, design flexibility, and aesthetics [2]. Fibre-reinforced polymers (FRPs) are among the most widely produced categories of composite materials. The dominant reinforcing fibres used in automotive, aerospace, and marine applications are carbon, glass, and kevlar [3]. In this framework, carbon-based (nano)materials have been widely applied in the fields of environmental science [4,5,6], food industry [7], new transportation solutions [8], medicine [9] and health applications [10,11], energy [12,13], construction [14], electronics, sports, and so on. In addition, they have gained considerable ground in high-performance applications, such as the aerospace [8,15], marine [16], military [17], and automobile industries [18]. Especially, the last decade has seen a very significant increase in the use of carbon fibre reinforced polymers (CFRPs) [19], as shown in Figure 1.

Global demand for carbon fibres (CF) is is expected to increase from 72,000 tonnes to 140,000 tonnes; the CFRP global profit is expected to increase from $28.2 billion to $48.7 billion [21].

As a result, these endless demands are exacerbating environmental issues such as global warming, climate change, natural resource depletion, waste disposal, loss of biodiversity, deforestation, ocean acidification, ozone layer depletion, acid rain, water pollution, and urban sprawl, which are responsible for environmental damage [22]. Furthermore, the production of CFs causes environmental hazards besides their high costs, which increased the interest in using more sustainable materials [21]. Carbon fibre reinforced polymers exhibit great potential among the other lightweight materials, nevertheless, CF manufacturing production generates about 15 times more CO_2_ than the conventional steel on a weight basis [23]. Moreover, virgin carbon fibres (vCFs) require significant energy during manufacturing—between 198 and 595 MJ/kg [24], approximately 10 times more than other synthetic fibres, such as glass fibres [25]. More specifically, CFs are produced by pyrolysis at 1000–1400 °C for high-modulus fibres or at 1800–2000 °C for high-strength fibres [26]. However, as manufacturing processes have progressed, energy consumption has decreased [27]. Much effort has been also applied on the functionalisation of CFs’ surface [28], in order to increase the fibre–matrix interface. However, the developed methods, based either on dry [29] or wet chemistry [30], contribute to the increase in environmental impacts, owing to the extra energy consumption and the chemicals used. Another aspect is that the use of recyclable polymers with carbon and other niche fibres renders the composite non-recyclable or, in some cases, non-profitable to be recycled [31]. As a consequence, it is considered a significant issue within the global community, while landfills are filling up at a faster pace along with the need for “going green” as a result of global warming [32]. Landfill is a fairly inexpensive method of disposal, however, based on European Union’s Waste Framework Directive, it is the least preferable management option [33]. In this context, the need to ensure the safety of the environment and the human health is required. Composite materials could fulfil the demands of sustainability. To tackle these issues, research in recent years has been focused on other eco-friendly options such as green synthesis, processing, applications, recycling, and biodegradation.

The life cycle assessment (LCA) is a vital tool at every stage of a product’s life stage from the initial synthesis to the final disposal in order to achieve a sustainable environment. LCA is a state-of-the-art, well-established, and ISO14044 standardised methodology that allows to take into account the entire life cycle of a product or system. By integrating the life perspective into the overall management and driving product and process production in a more sustainable direction, the developer can achieve environmental, occupational health and safety, risk and quality control, as well as green processes and product options [34]. Thus, LCA provides a comprehensive framework for a holistic environmental assessment, which is particularly useful to prevent burden shifting across the life cycle stages (e.g., when lower impacts during the use stage are counterbalanced by higher impacts during production) or across impact categories (e.g., savings in climate change, but burdens regarding resource consumption—ISO 14001) [35,36]. Across the life cycle, LCA makes an inventory of all technical processes and use of resources required to deliver the service provided by the assessed product or system. Then, LCA identifies environmental exchanges (emissions of substances and wastes) occurring to different environmental compartments (like the air, water, and soil) and further translates these exchanges into environmental impacts (such as climate change, ozone depletion, and toxicity to ecosystems and humans).

Regarding nanofibres, the potential human toxicity and ecotoxicity are of major concern [37]. While they are potentially less dangerous as inclusions, free particles within the range of nanometer increase health and environmental concerns owing to their high surface area to mass ratios and their ability to penetrate biological cells [37,38]. Carbon nanofibres could exhibit toxic properties similar to those of asbestos however, because of data scarcity, this effect has not yet been considered in LCA studies.

As the European Union (EU) circular economy strategy targets sustainable products in the market to minimize waste, while EU sets a clear vision for climate neutrality by 2050 [39], viable waste management solutions need to be defined for composites. The circular economy plan of the European Commission aims to raise recycling rates and reduce the amount of urban waste that could go to landfill by 2030 to 10% [40]. In addition, polymer waste management should comply with the existing European Directives and regulations, such as the Landfilling Directive [41], the Packaging Waste Directive [42,43], and the Directives on end-of-life on vehicles (2000/53/EC, 2000), on (waste) batteries and accumulators [44] and on waste electrical and electronic equipment [45]. However, it is not yet clear how this would influence the industrially derived and non-packaging construction waste (of which 75% must be recycled by 2030 [46]). The emissions target for passenger cars [47] sets a maximum emission limit of 95 g CO_2_/km, averaged by 2020 across a range of manufacturers. This goal is unlikely to be realised just with powertrain and aerodynamic improvements. In order to meet this target, the reduction of vehicle weight with the use of composite materials is crucial. Although, the End-of-Life Vehicle Directive (ELV) [43] requires 85% by weight of vehicles to be reused or recycled, and 95% to be reused, recycled, or recovered (including burnt for energy recovery). CFRP composites provide the greatest potential for weight decrease, but their current costs [48] and the lack of a sustainable recycling route are the main challenges to CFRP uptake [49]. The global CFRP waste is expected to be increased at 20 kt annually by 2025. Approximately 6000–8000 commercial aircraft will be approaching their end-of-life by 2030, hence the high demand for CFRPs to establish economically viable waste management and recycling technology [50]. To date, LCA has been employed to characterise the environmental performance of novel CFRPs matrix composite materials in such sectors [51]. Research findings highlight the importance of the end-of-life stage in the overall environmental performance [52], as recyclability aims significantly to consider avoidance (or at least partial replacement) of virgin raw material production with corresponding environmental savings. It is also aligned to the EU policy mandate for transitioning towards a circular economy [53]; nonetheless, the quality of the secondary product (recycled CFRP—rCFRP) is still unclarified.

From the available technologies, pyrolysis has been described as the most promising recycling solution for CFRPs, in terms of efficiency [54]. However, several approaches have been also developed to reclaim CFs from CFRPs such as the following [31,55,56]: mechanical recycling, incineration, thermal pyrolysis and fluidized bed process, chemical recycling based on supercritical solvents, and solvolysis. It should be mentioned that these approaches are rather energy intensive, in addition to releasing highly toxic gases [54].

Currently, landfill and incineration with energy recovery (approximately 30 MJ/kg [57]) are the two commonly used waste treatment methods concerning CFRPs [58]. Both methods are not considered as recycling processes, as they do not incorporate the recovery operation. According to the recent literature, 38.4 MJ/kg is consumed in order for CFRP to be recycled with the chemical method [25]; this is equivalent to 10~30% of the total energy required to manufacture virgin fibres. The energy footprints of the chemical recycling in comparison with the pyrolysis is six time smaller, while the greenhouse gas (GHG) emissions are 1196.22 g CO_2_-eq and 5916.08 g CO_2_-eq for chemical recycling and pyrolysis, respectively [59,60]. Moreover, despite the fact that mechanical recycling is considered as a cost-effective process, the mechanical performance of the reclaimed chopped fibres is significant low, because of the lack of control in terms of length, length distribution, surface quality (adhesion into the novel composite), or origin (different producers) [31]. The energy required to recycle CF by the mechanical method (0.27–2.03 MJ/kg) is lower in comparison with the energy required to manufacture virgin CF (183–286 MJ/kg). In addition, using pyrolysis and fluidized bed processes, only 70–75% of the strength retention in the reclaimed fibres is recovered [54]. Thermal recycling methods for CFRP include a high temperature in order to break down the matrix fibre and obtain the fibres. These methods are known as pyrolysis and fluidized bed recycling or their variations. Th pyrolysis process has an energy consumption of around 30 MJ/kg CFRP and is considered average compared with the other widely adopted industrial processes [54]. Recycling of CFRPs is also influenced by the polymer matrix properties; thermoset polymers are mainly used as matrix for structural CFRPs, representing around 80% of polymer matrix composites. The solvolysis process has a 78 times greater human health impact, 76 times greater ecotoxicity, 17 times greater carbon footprint (global warming), and 3 times greater ozone depletion in comparison with pyrolysis [61]. The size-decrease alternative method using high voltages, although not being energy efficient (requires 19 MJ/kg for fibre recovery), shows a strong potential for future growth because it allows for the recovery and reuse of organic resinous products [62]. Such methods are the electrodynamical fragmentation (EDF), in which a high voltage pulse (50–200 kV) passes into ionised water and decomposes the CFRP waste into smaller parts; and high voltage fragmentation (HVF) with a high voltage pulse (160 kV), which also decomposes the CFRP waste into smaller parts. In both methods, clean and long fibres are received [63]. In Table 1, the energy required for the recycling of 1 kg CFRP waste using different recycling routes is summarised.

Overall, the high demand of lightweight products causes the wide use of CFRP matrix composites; however, energy intensity, cost, and final disposal remain major barriers to the wide adaptation of these materials for industrial applications. In addition, it has to be mentioned that nanoadditivated composites make LCA rather difficult because of data scarcity, as well as inherent composite properties such as good mechanical robustness and stability; this makes detachment, sorting, separation, and so on more difficult when seeking recycling/repurposing. In this study, recycling scenarios are discussed as environmental preferable options for global warming potential (GWP) regarding CFRP wastes, indicating the potential benefits of CFRP recycling in comparison with other disposal routes. By performing an LCA of the new materials, this study aims to investigate what are the environmental hotspots for further environmental improvements. It also aims to showcase the usefulness of LCA as a tool for sustainable innovation in new product development. The suggested perspective aims to assist stakeholders (e.g., producers and policy makers) in making more informed decisions, for example, for better performance regarding environmental performance and better quality control [34]. CFRP has become the preferred choice of material in sectors such as sporting goods and automotive thanks to its lightweight and high-performance properties. However, the rapid development of CFRP will result in increased CFRP wastes disposal in landfills that takes no advantage of the residual value, but adds burden to waste management [21]. In order to address this problem, various recycling methods, such as mechanical recycling and pyrolysis, have been compared in this study, providing recycling recommendations for decision making in industries. Two differed cases have been studied, (a) SleekFast sailing boat and (b) handbrake lever, for motorcycles. Both were manufactured with new developed carbon-based composite materials using different production technologies (hand layup and vacuum bagging and direct infusion molding process, respectively).

## 2. Methodology

For the description of the methodology and LCA calculation as such, the International Reference Life Cycle Data System (ILCD) Handbook suggested by the European Commission [66] is followed.

### 2.1. Goal and Scope

As a first step, the goal (i.e., the purpose) and scope (i.e., what to analyse and how) were identified. The goals of the LCAs and the intended applications coincide with the research objectives presented in the introduction. In terms of scope, the functional unit (FU), which reflects the primary function of the system, was defined as “1 piece SleekFast sailing boat 13.5 ft long and 5.5 ft wide” with a lifetime of 30 years; and a “1 piece of handbrake lever” for motorcycles with a lifetime of 20 years.

**Figure 2:**
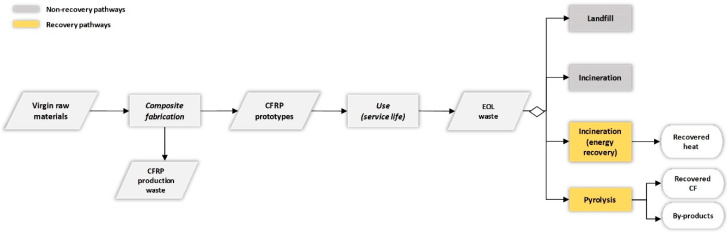
System boundaries considered for both CFRP prototypes: the SleekFast sailing boat and the handbrake lever. EOL, end-of-life.

The system boundaries (Figure 2) consist of the life cycle of the products from the extraction of raw materials to the end-of-life (EoL). As these are descriptive studies aiming to document the analysed systems, the modelling principle for the life cycle inventory followed an attributional LCA approach [66], while multi-functionality of processes is addressed by use of system expansion. For example, in the waste treatment stage, incineration and recycling lead to energy and materials recovery, avoiding respective production from virgin sources.

### 2.2. Inventory Data Collection

To ensure technological and market representativeness, primary data were collected from both material developers and industry. Detailed accounts of primary inventory data are given in Supporting Information (Appendix A), while the aggregated inventory is given in Table 2 per life cycle stage. In order to model the background processes, for example, extraction of the materials, waste treatment, and so on, the study relied on generic data from ecoinvent v3.4 without technological adjustment [67].

#### 2.2.1. SleekFast Sailing Boat Case

Raw materials included modified SICOMIN SR1500 epoxy resin, with multiwall carbon nanotubes (MWCNTs), and piles of commercial CF fabric that has undergone electropolymerisation with polymethacrylic acid, according to the procedure described by [30] and [68], in order to increase the affinity of the fibres with the matrix and to enhance the mechanical properties of the structure. The boat has been manufactured with the hand-layup technique. Production scrap during the manufacturing stage of the composite prototype was set at 6% of the total mass. This is in line with literature studies, recognizing that production scrap can reach up to 10% [69]. For the use stage, a 50% service life extension has been assumed, while there is no repairing requirement. This is a realistic assumption based on typical use stage conditions (intensive care practice for the specific boat type). For maintenance, the assumption is that there is a need for 30 kg of epoxy gel coat (2 kg biannually).

#### 2.2.2. Handbrake Lever for Motorcycle Case

Raw materials included epoxy resin and CNT deposited on CF fabric (C-Weave 400T 6K HS X125CM) via electrophoretic deposition (EPD). The handbrake lever was manufactured by hand layup followed by wet compression moulding. Production scrap during the manufacturing stage of the composite prototype was assumed as 2% (as trimming waste) owing to the small size of handbrake lever. The use phase in the case of handbrake lever was not taken into consideration. However, the service life time of the handbrake lever would coincide with the operational life time of the motorcycle.

#### 2.2.3. Assumptions Applicable for Both Test Cases

For the carbon fibre production, representative data of industrial process including polyacrylonitrile (PAN) production and relative flows are used [70]. As Japan is the main global supplier of CF and PAN precursor production [71], the corresponding production processes were modelled using the Japanese electricity grid mix, because this is where 70% of global production takes place [72]. A prior pre-treatment to reduce the size of composite waste is required before any other waste treatment, thus an industrial shredder consuming 0.27 MJ/kg was assumed [55]. As CFRP prototypes have been demonstrated by consortium partners located in Europe, energy for recycling facilities (pyrolyzer) is modelled using the EU-27 electricity grid mix. Landfill is considered as the reference EOL, as it is the dominant waste management pathway to date [73,74].

### 2.3. Scenario Analysis for the End of Life Stage

**Figure 3:**
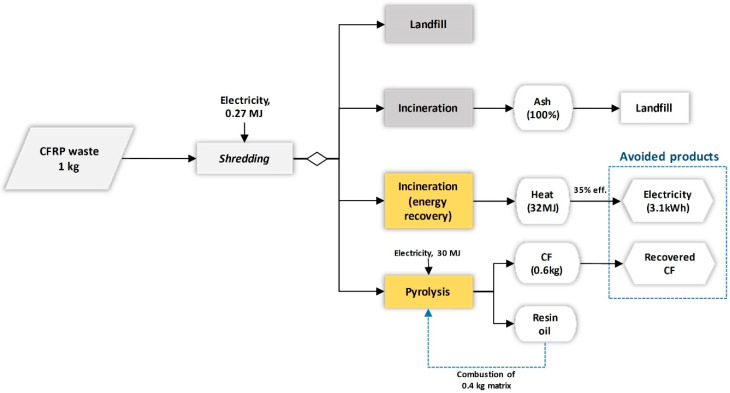
End-of-life (EOL) treatment scenarios for the composite products (values given per kg product waste).

The EoL of composite materials is associated with uncertainties due to temporal aspects (long life time) and the difficulty in predicting future markets. Uncertainty is enhanced by the lack of inventory data for technologies such as recycling of carbon fibres. Policy wise, in the EU, the political targets such as those set by the Waste Framework Directive aim at high recycling rates, for example, there was the unmet target for 70% recycling of non-hazardous construction and demolition (C&D) waste by 2020 [79]. Thus, researchers have concluded on pyrolysis of CFPR waste management [75] [80,81] and, experimentally, solvolysis [82,83,84] as the most promising recycling options up to date. In addition, the high value of CFs and the tightening legislation on disposal were also to enhance this initiative [85]. Given both the uncertainty of the EoL stage and its potential for improving the overall environmental performance of the CFRP, the reference EoL scenario was compared with three alternative EoL scenarios, as shown in Figure 3 and Table 3.

### 2.4. Impact Assessment Indicators

**Figure 4:**
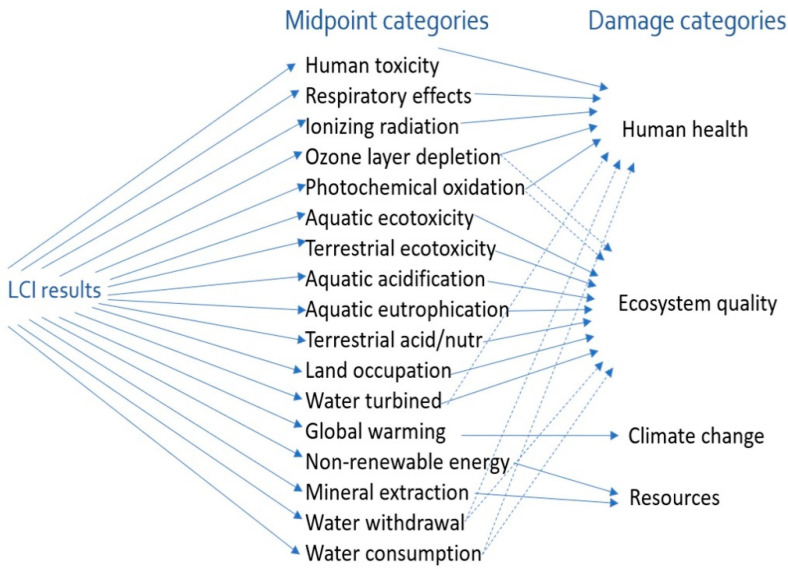
Mid- and end-point environmental impact categories assessed [91]. LCI, life cycle inventory.

Of primary interest was the system’s contribution to climate change. This was assessed via the IPCC findings [87]. In addition to climate change, all ILCD 2011 Midpoint+ recommended impact categories [88] at a midpoint level were assessed (see Figure 4). The systems were modelled in SimaPro software 8.0.4.26, which is one of the commercial LCA software packages. Uncertainties due to the combined effect of software and database choices are expected to influence the final results, however, they are not expected to change the conclusions made based on the primary environmental indicator selected (carbon footprint) [89,90].

## 3. Results and Discussion

**Figure 5:**
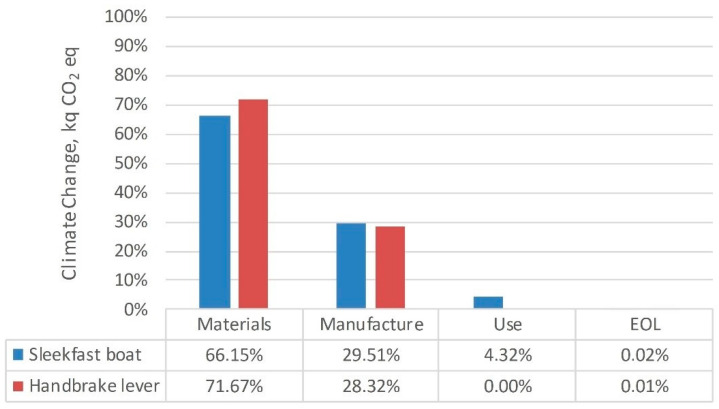
Percent contribution of life cycle stages to the total impact for the two case studies: composite Sleekfast sailing boat and handbrake lever.

The LCA results were used to identify hotspots in two dimensions: across the life cycle of the product and within each life cycle stage to the maximum resolution level according to the available data. As Figure 5 shows for climate change, most of the impacts are due to extraction and production of materials (hereafter called ‘materials’). This stage contributes more than 65% and 70% to the climate change impact for the boat and the lever case, respectively, followed by the manufacture stage (approximately 30% for both studied cases). These results are in line with relevant work of the literature [92,93,94]. The reference EOL scenario has a minor contribution to climate change, mainly owing to the assumption that composite waste behaves as inert material in a landfill, thus a negligible contribution to methane emission is considered over the years [95,96].

**Figure 6:**
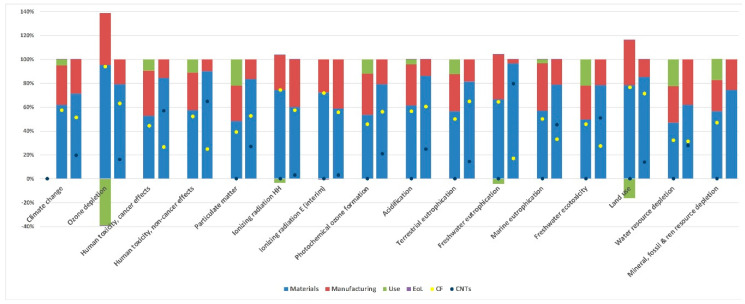
Percent contribution of the life cycle stages and of the main raw materials for the two studied cases. CNT, carbon nanotube.

As Figure 6 shows for both cases, ‘materials’ is the most relevant life cycle stage across impact categories, accounting for 47–72% of the life cycle impacts for the case of the Sleekfast boat and 60–90% for the case of the handbrake lever. The main contributing factors within the ‘materials’ life cycle stage are presented in Figure 5. For the Sleekfast boat, CF is consistently an environmental hotspot, accounting for 32–71% of the ‘materials’ impact. For the handbrake lever, CNTs are also a hotspot and, for some impact categories, such as the ones related to toxicity, the contribution of CNT is higher than that of CF. Detailed accounts of the contribution of each inventory aspect to each impact category are given in Table A3 and Table A4 in Appendix A.

**Figure 7:**
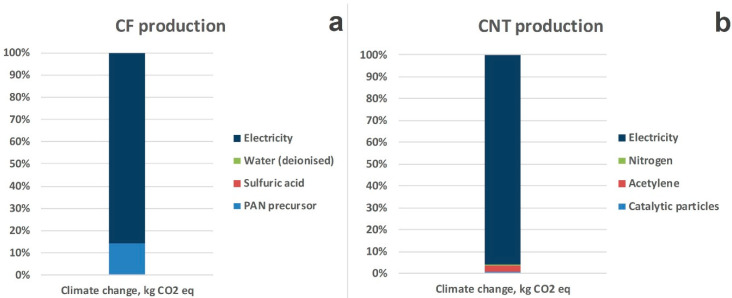
Percent contribution of CF (**a**) and CNT (**b**) production on climate change impact category. PAN, polyacrylonitrile.

Focusing on the two main impact contributors, CFs are manufactured from a polymeric feedstock, the “precursor”, which in majority is derived from polyacrylonitrile (PAN). Precursor formulation (polymerization) begins with an acrylonitrile monomer, which is combined in a reactor with plasticized acrylic co-monomers and a catalyst (sulfuric acid). As Figure 7 indicates for climate change, CF production is a highly energy-intensive process (E = 368 MJ/kg CF), with electricity accounting for 85% on climate change impact and PAN precursor for 15% (for the production of 1 kg PAN precursor, acrylonitrile has the major contribution, 53% over 47% of electricity, on climate change impact). As for CNTs, the electricity requirements for their production are also the major hotspot [97].

As the productions of carbon fibres and CNTs are energy intensive processes, the GHG emissions highly depend on the electricity grid source. Indicatively, the CO_2_ intensity of electricity generation in EU-27 is 0.295 gCO_2_/kWh [98], and in Japan, it is 0.496 g CO_2_/kWh [99]. This is expected as the Japanese grid has a renewable energy share of 16% [100], while the share in EU-27 is 29% [101]. Aside from climate change, electricity generation gives rise to negative impacts on the environment and human health throughout all life cycle stages.

### End of Life–The Most Uncertain Life Cycle Stage with the Highest Potential for Improvement

**Figure 8:**
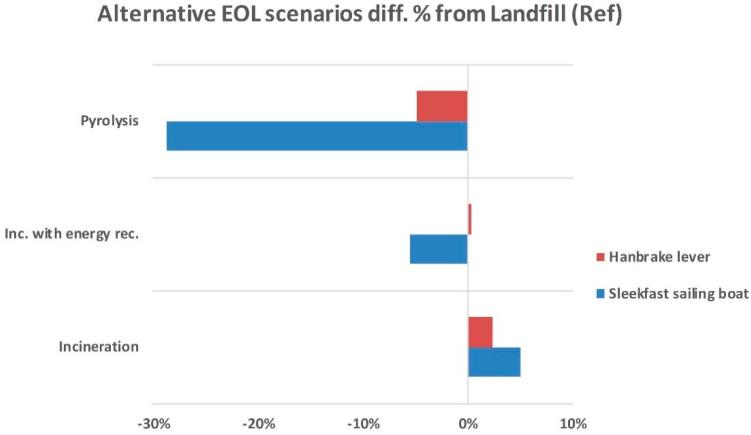
Percentage difference in climate change impact of the alternative EOL scenarios compared with the reference.

Given the domination of materials in the overall impacts, their treatment and recycling at the EoL of the plants is consequently important as it could lead to environmental savings due to the avoided production of raw materials (recycled carbon fibres) through system expansion (Figure 8). The results of the scenario analysis for the different EoL pathways indicated that the savings from pyrolysis can contribute up to 40% in the total impact. The results, aligned with other literature, signify the potential environmental improvements due to recovery and recycling.

## 4. Discussion

Salt and corrosive water can cause severe damages to watercrafts over time, while thermoset composites could be an attractive substitute over the predominantly used materials (GRP) thanks to their strong resistance in corrosion and fatigue, also minimizing the maintenance requirements [102]. The wide development of reinforced epoxies in the last decades can offer significant performance and quality improvements to the boat as a result of higher elongation, tensile strength, and modulus over polyester (PE) and vinyl ester (VE) resins; CFRP use in marine applications in turn gives technical advantages to shipbuilders. Reduced weight is achieved by increasing the fibre content while decreasing the number of layers without compromising the mechanical behaviour of the hull, thus providing higher speed and less fuel consumption. In addition, because of the high value of CF, research has continued to focus on improving recyclability to recover CF from CFRPs via thermal and chemical techniques [31]. Despite the fact that is a domain rapidly advancing and that carbon fibre/epoxy composites are increased providing materials in many industries (automotive, manufacturing, aerospace), the enormous generation of CFRP wastes within few years remains an unsolved problem. Recently, several recycling pathways of carbon fibres are reaching a mature phase, as recovered CF from waste can be achieved at one order of magnitude (€5/kg, [25]) less than virgin CF cost; vCF prices are estimated at €30–60/kg, [103]. The energy required to recycle CF via pyrolysis is 5–10% less compared with the energy for virgin CF production (13–32 MJ/kg and 183–286 MJ/kg, respectively) [104]. Additionally, initiatives from large aerospace companies (e.g., Airbus) promote environmental responsibility across the entire lifecycle of an aircraft. The cost savings of recycled fibres can generally be 20–40% [49]. This constitutes recycled carbon fibres as tentative substitutes of glass fibre or virgin carbon fibre in applications with comparable mechanical properties.

**Figure 9:**
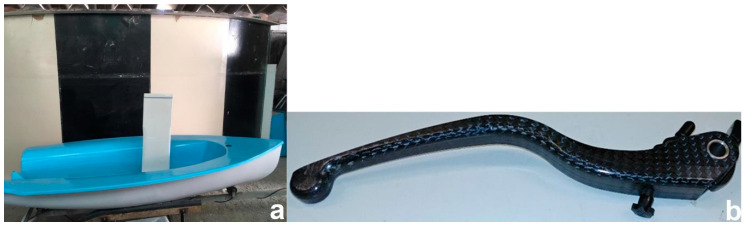
MODCOMP project advanced material prototypes: (**a**) sailing boat; (**b**) lever.

CFRP recycling and the reuse of the recovered CF in composite materials (e.g., Figure 9) offer low production impacts associated with the virgin CF substitution, while the respective low cost could open up new composites markets—for example, in the automotive sector [96]. While the direct life cycle environmental impact of a composite is dependent on the design criteria of the components, recycled CF components can provide low production impacts relative to lightweight competitor materials (e.g., aluminium, virgin CFRP) [25].

Vo Dong et al. [76] performed an economical and environmental assessment on the recovery and disposal pathways for CFRP waste management and indicated that the carbon fibre recycling cannot currently compete in cost with the conventional landfill and incineration routes. It was estimated that disposal of CFRP waste would require approximately 0.1 €/kg, without accounting profits from recoverable products in waste. Thus, recycling of carbon fibres needs to reduce further the unit cost to be comparable with current non-recover pathways. To conclude, fibre recovery rate and recycling capacity are pivotal to decrease the unit cost of recycled fibre as well as GWP impacts.

Even if LCA is considered a holistic, comprehensive methodology for environmental impact assessments, it still lacks the capacity to assess the impact of nanomaterials and nano-enabled products. Despite the efforts [105,106], the potential impacts of the released NMs to human and environmental health have not been covered yet, while several uncertainties and data gaps exist [107,108]. Another issue is that nanomaterials, which are increasing in number in several everyday applications the last decade, are entering into waste streams as part of end-of-life products along with conventional waste, without any real understanding of their environmental impacts or health risks on human beings and living organisms [109]. Moreover, concerning the EoL of NMs, there are only few studies that have investigated the presence of NMs’ emissions in municipal solid waste (MSW) incineration. For instance, in the case of CNTs, there are contradictory findings; from an efficiency burn at approximately 94%, as a result of their chemical nature, to the opposite scenario of a non-efficient incineration. Thus, further investigation into the different NMs types and treatment routes is required [109].

## 5. Conclusions

In the present study, the environmental impacts over the entire life cycle of two carbon fibre/epoxy composite prototypes were assessed, assuming four end-of-life treatments for CFRP waste to be hypothetically performed; namely, (a) landfill, (b) incineration of hazardous waste, (c) incineration with energy recovery, and (d) thermal recycling via pyrolysis. The former technology has been set as the reference scenario with which to be compared. The functional unit was 1 piece of carbon fibre/epoxy composite prototypes. The results of this work have indicated that there are potential benefits from recycling of CFRP waste that are strongly associated with the environmental impacts of the materials. These include the following:

1. ‘Materials’ contribution dominates in the calculated 14 impact categories, with CF and CNTs being the environmental hotspots, both thanks to the high energy intensive production process. The laboratory production of CNTs required 1104 MJ/kg CNTs. While the upscale of CNT manufacturing becomes more mature reaching the industrial-scale, a reduction of approximately two to three orders of magnitude in manufacturing energy intensity can be expected [110].
2. Recycling via pyrolysis can lead to significant impact savings due to material recovery (up to 40% for the case of Sleekfast boat).
3. Incineration with energy recovery also leads to environmental savings, but much lower than recycling (~7 and ~4 times lower for the boat and the lever cases, respectively).
4. Incineration without energy recovery contributes negatively by approximately 5% and 2% to climate change for the boat and lever, respectively. It is worth mentioning that this option accounts for around 1–2% of total municipal waste treated inside and outside Europe [111].

The use of the LCA tool provided insights into the environmental performance of processes across the life cycle stage of a CFRP product, allowing for product developers to set the focus right on future environmental improvements and eco-design of novel carbon fibre-based materials. The results showcased that carbon fibre and CNTs production are energy-intensive processes and the main contributors to climate change. Thus, energy efficiency measures and cleaner electricity grid mixes can have a significant influence on the environmental performance. Aligning with other literature, the study also showed that research efforts should focus on improving recycling technologies to recover high quality CF from CFRP waste and reuse them in manufacturing high performance materials, ideally of equal virgin CFRP performance.

## Figures and Tables

**Table 1:** Energy required (MJ) in order to recycle 1 kg carbon fibre reinforced polymer (CFRP) using different routes.

CFRP Recycling Routes	Energy Consumption MJ/kg CFRP	Reference
Mechanical	0.17–1.93	[64]
Mechanical	0.27–2.03	[55]
Pyrolysis	3–30	[64]
Solvolysis	19.2	[62]
Chemical	60–90	[65]

**Table 2:** Aggregated inventory per life cycle stage and related assumptions *. CF, carbon fibre; MWCNT, multi-walled carbon nanotube; MSW, municipal solid waste.

	Materials	Manufacture	Use	End-of-life
*Sleekfast sailing boat*	**Input** CF = 10.5 kg Sicomin SR1500 (0.06 wt% MWCNTs) = 10 kg PVC foam = 1.5 kg Epoxy gelcoat = 4 kg Electricity = 368 MJ/kg CF Electricity = 1104 MJ/kg CNTs **Output/emissions to air/water** (see Table A2 in Appendix A)	**Input** Auxiliaries = 2.72 kg Scraps = 1.47 kg (6%) Electricity = 230 MJ (curing) *Manufacture waste* - Scrap waste (6%) hazardous incineration - Aux. waste (96%) hazardous incineration and 4% recycling of plastic	**Input** Epoxy gelcoat = 30 kg (for maintenance, 2 kg biannually for 30 years’ lifetime)	***Scenario 1: Landfill*** **Input** 100% inert waste [75] Electricity = 0.27 MJ/kg ***Scenario 2: Hazardous waste incineration*** **Input** Electricity = 0.27 MJ/kg ***Scenario 3: MSW incineration with energy recovery*** **Input** Electricity = 0.27 MJ/kg **Avoided products** Electricity = 3.1 kWh [76] Heat for reuse = 32 MJ/kg [77] ***Scenario 4: Recycling*via *pyrolysis*** **Input** Electricity = 0.27 MJ/kg Electricity = 30 MJ/kg [61] **Avoided products** CF recovery 60% [78] Heating oil = 30 MJ/kg [77]
*Handbrake lever*	**Input** CF = 0.12 kg Epoxy resin = 0.02 kg CNTs = 0.01 kg Electricity = 368 MJ/kg CF Electricity = 1.1 MJ/kg CNTs **Output/emissions to air/water** (see Table A1 in Appendix)	**Input** Pack. mat. = 1.272 kg Electricity = 18 MJ (curing) *Manufacture waste* - Scrap waste (2%) treated as MSW (incineration) - Pack. waste (100%) is recycled	**Assumptions**: - service lifetime of handbrake lever equals to motorcycle lifetime - no maintenance - no repairing	

* More details are provided in Table A1 and Table A2 in Appendix A.

**Table 3:** The four end-of-life (EoL) scenarios and the respective waste treatment technology applied.

Scenario	Waste Management	Waste Treatment Technology	Comments
**Scenario 1**	Landfill	Sanitary landfill	100% inert waste [75,86]
**Scenario 2**	Incineration of hazardous waste	Rotary kiln (>1100 °C)	100% bottom ash sent to hazardous landfill
**Scenario 3**	Incineration with energy recovery	MSW incinerator (850 °C–1200 °C)	35% heat recovery convert to electricity [75]
**Scenario 4**	Thermal recycling	Pyrolysis (450 °C and 700 °C)	60% CF recovery and retrieved energy of 40 MJ/kg matrix [75]

## Appendix A

**Table A1:** Life cycle inventory (LCI) of 1-piece (0.15 kg) handbrake lever.

**MATERIALS**		
**Input (materials, energy, etc.)**	**Unit**	**Comment**
Carbon fibre	0.12 kg	[70] (updated 2014)
Epoxy resin {GLO}	0.02 kg	Ecoinvent 3.3
CNTs	0.01 kg	Experimental Data
Electricity, medium voltage {GR}	11.04 MJ	CNT production energy, experimental Data
Electricity, medium voltage {JP}	45.631 MJ	CF production energy [70,112]
**Output/Emissions to air**	**Unit**	**Comment**
Ammonia	0.0001 kg	[70] (updated 2014)
Hydrogen cyanide	0.0019 kg	[70] (updated 2014)
Carbon monoxide	0.0004 kg	[70] (updated 2014)
Carbon dioxide	0.1216 kg	[70] (updated 2014)
Ethane	1.2 × 10^−6^ kg	[70] (updated 2014)
Acrylonitrile	0.0026 kg	[70] (updated 2014)
Nitrogen	0.2144 kg	Experimental Data
VOC, volatile organic compounds	0.0053 kg	Experimental Data
Soot	0.0013 kg	Experimental Data
Ethyne	0.0073 kg	Experimental Data
**Output/Emissions to water**	**Unit**	**Comment**
Sulfuric acid	0.0024 kg	[70] (updated 2014)
N-N-Dimethylformamide	0.0021 kg	[70] (updated 2014)
PAH, polycyclic aromatic hydrocarbons	6.6 × 10^−5^ kg	Experimental Data
**MANUFACTURE**		
**Input (materials, energy, etc.)**	**Unit**	**Comment**
Production scrap	0.02 pc	Assumed 2% production scrap
Packaging materials	1.272 kg	Experimental Data
Electricity, medium voltage {EU-27}	18 MJ	Experimental Data
**EOL DISPOSAL**		
**Scenario 1: Landfill**	**Unit**	**Comment**
Inert waste	0.15 kg	[75]
Electricity, medium voltage {EU-27}	0.0405 MJ	Industrial shredder energy [55]
**Scenario 2: Incineration of Hazardous waste**	**Unit**	**Comment**
Electricity, medium voltage {EU-27}	0.0405 MJ	Industrial shredder [55]
**Scenario 3: MSW incineration with energy recovery**	**Unit**	**Comment**
Electricity, medium voltage {EU-27}	0.0405 MJ	Industrial shredder [55]
**Output/ avoided products**	**Unit**	**Comment**
Electricity, medium voltage {EU-27}	1.68 MJ	35% efficiency [112]
Heat, for reuse in municipal waste incineration {EU-27}	4.8 MJ	[81] [75]
**Scenario 4: Thermal recycling via pyrolysis**	**Unit**	**Comment**
Electricity, medium voltage {EU-27}	0.0405 MJ	Industrial shredder [55]
Electricity, medium voltage {EU-27}	4.5 MJ	[61]
**Output/ avoided products**	**Unit**	**Comment**
Carbon fibre	0.0612 kg	60% CF recovery [78]
Electricity, medium voltage {EU-27}	5.94 MJ	[77]

**Table A2:** LCI of 1-piece (26 kg) ‘Sleekfast’ sailing boat.

**MATERIALS**		
**Input (materials, energy)**	**Unit**	**Comment**
Carbon fibre	10.5 kg	[70] (updated 2014)
SICOMIN 1500 (0,06 wt% MWCNTs)	10 kg	Experimental Data
PVC foam	1.5 kg	Ecoinvent 3.4
Epoxy gelcoat	4 kg	EuCIA - EcoImpact Calculator report v1.3, Ecoinvent 3.4
Electricity, medium voltage {JP}	3680 MJ	CF production energy [70,112]
Electricity, medium voltage {EU-27}	6.48 MJ	Experimental Data
Electricity, medium voltage {PT}	600 MJ	Experimental Data
**Output/Emissions to air**	**Unit**	**Comment**
Ammonia	0.0122 kg	[70] (updated 2014)
Hydrogen cyanide	0.1649 kg	[70] (updated 2014)
Carbon monoxide	0.0340 kg	[70] (updated 2014)
Carbon dioxide	10.636 kg	[70] (updated 2014)
Ethane	0.0001 kg	[70] (updated 2014)
Acrylonitrile	0.2243 kg	[70] (updated 2014)
Nitrogen	0.1415 kg	Experimental Data
VOC, volatile organic compounds	0.0035 kg	Experimental Data
Soot	0.0009 kg	Experimental Data
Ethyne	0.0048 kg	Experimental Data
VOC, volatile organic compounds	0.0196 kg	Experimental Data
Carbon dioxide	3.040 kg	Experimental Data
**Output/Emissions to water**	**Unit**	**Comment**
Sulfuric acid	0.2089 kg	[70] (updated 2014)
N-N-Dimethylformamide	0.1857 kg	[70] (updated 2014)
PAH, polycyclic aromatic hydrocarbons	4.4 × 10^−5^ kg	Experimental Data
**MANUFACTURE**		
**Input (materials, energy)**	**Unit**	**Comment**
Production scrap	1.47 kg	Experimental Data
Packaging film, low density polyethylene {GLO}	0.35 kg	Experimental Data
Acetone, liquid {GLO}	2.37 kg	Experimental Data
Electricity, medium voltage {PT}	230.4 MJ	Experimental Data
**USE**		
Epoxy gelcoat	30 kg	Assumed 2 kg biannually for 30 years operational life (EuCIA - EcoImpact Calculator report v1.3, Ecoinvent 3.4)
**EOL DISPOSAL**		
**Scenario 1: Landfill**	**Unit**	**Comment**
Inert waste	26 kg	[75]
Electricity, medium voltage {EU-27}	7.02 MJ	Industrial shredder energy [55]
**Scenario 2: Incineration of Hazardous waste**	**Unit**	**Comment**
Electricity, medium voltage {EU-27}	7.02 MJ	Industrial shredder [55], Incineration Haz. (Ecoinvent)
**Scenario 3: MSW incineration with energy recovery**	**Unit**	**Comment**
Electricity, medium voltage {EU-27}	7.02 MJ	Industrial shredder [55]
**Output/ avoided products**	**Unit**	**Comment**
Electricity, medium voltage {EU-27}	291 MJ	35% efficiency
Heat, for reuse in municipal waste incineration {EU-27}	832 MJ	[75,81]
**Scenario 4: Thermal recycling via pyrolysis**	**Unit**	**Comment**
Electricity, medium voltage {EU-27}	7.02 MJ	Industrial shredder [55]
Electricity, medium voltage {EU-27}	780 MJ	[61]
**Output/ avoided products**	**Unit**	**Comment**
Carbon fibre	6.3 kg	60% CF recovery [78]
Electricity, medium voltage {EU-27}	396 MJ	[77]

**Table A3:** Percentage contribution of each inventory aspect to each impact category for the Sleekfast sailing boat.

Impact Category	Unit	Materials	Manufacturing	Use	EoL	CF in Materials
Climate change	kg CO_2_ eq	62%	33%	5%	0%	57%
Ozone depletion	kg CFC-11 eq	54%	24%	22%	0%	53%
Human toxicity, cancer effects	CTUh	53%	38%	9%	0%	44%
Human toxicity, non-cancer effects	CTUh	57%	31%	11%	0%	52%
Particulate matter	kg PM2.5 eq	48%	30%	22%	0%	39%
Ionizing radiation HH	kBq U235 eq	70%	27%	3%	0%	69%
Ionizing radiation E (interim)	CTUe	72%	28%	0%	0%	71%
Photochemical ozone formation	kg NMVOC eq	53%	34%	12%	0%	45%
Acidification	molc H+ eq	62%	34%	4%	0%	57%
Terrestrial eutrophication	molc N eq	56%	31%	13%	0%	50%
Freshwater eutrophication	kg P eq	62%	35%	4%	0%	59%
Marine eutrophication	kg N eq	57%	40%	3%	0%	50%
Freshwater ecotoxicity	CTUe	50%	28%	22%	0%	46%
Land use	kg C deficit	59%	29%	12%	0%	57%
Water resource depletion	m^3^ water eq	47%	30%	23%	0%	32%
Mineral, fossil, and ren resource depletion	kg Sb eq	56%	26%	17%	0%	46%

**Table A4:** Percentage contribution of each inventory aspect to each impact category for the handbrake lever.

Impact Category	Unit	Materials	Manufacturing	EoL	CF in Materials
Climate change	kg CO_2_ eq	72%	28%	0%	51%
Ozone depletion	kg CFC-11 eq	79%	21%	0%	63%
Human toxicity, cancer effects	CTUh	84%	16%	0%	27%
Human toxicity, non-cancer effects	CTUh	90%	10%	0%	25%
Particulate matter	kg PM2.5 eq	83%	17%	0%	53%
Ionizing radiation HH	kBq U235 eq	60%	40%	0%	57%
Ionizing radiation E (interim)	CTUe	59%	41%	0%	56%
Photochemical ozone formation	kg NMVOC eq	79%	21%	0%	56%
Acidification	molc H+ eq	86%	14%	0%	60%
Terrestrial eutrophication	molc N eq	82%	18%	0%	65%
Freshwater eutrophication	kg P eq	97%	3%	0%	17%
Marine eutrophication	kg N eq	79%	21%	0%	33%
Freshwater ecotoxicity	CTUe	79%	21%	0%	28%
Land use	kg C deficit	85%	15%	0%	71%
Water resource depletion	m^3^ water eq	62%	38%	0%	31%
Mineral, fossil, and ren resource depletion	kg Sb eq	74%	26%	0%	60%
